# Development and validation of a nomogram for predicting early stress urinary incontinence following endoscopic enucleation of the prostate

**DOI:** 10.1007/s00345-021-03592-x

**Published:** 2021-01-21

**Authors:** Xuanhao Li, Fei He, Cong Huang, Liangshuo Zhang, Qiang Liu, Jian Song

**Affiliations:** 1 Department of Urology, Beijing Friendship Hospital, Capital Medical University, Beijing, People’s Republic of China; 2 Department of Urology, Xinhua Hospital, Shanghai Jiaotong University, School of Medicine, Shanghai, People’s Republic of China

**Keywords:** Stress urinary incontinence, Endoscopic enucleation of the prostate, Predictive model

## Abstract

**Purpose:**

To develop and validate a predictive nomogram for early stress urinary incontinence (SUI) after endoscopic enucleation of the prostate (EEP) in patients with benign prostatic hyperplasia (BPH).

**Methods:**

The records of 458 patients who underwent plasmakinetic- or diode-based EEP at our center from March 2016 to December 2019 were reviewed. Among these, 326 and 132 cases were randomly assigned to the training and validation set, respectively. A predictive nomogram was constructed based on multivariate logistic regression analysis. Receiver operating characteristic (ROC) analysis and calibration curves were employed to evaluate its performance.

**Results:**

65 years ≤ age < 70 years, 75 years ≤ age, 25 kg/m^2^ ≤ BMI < 30 kg/m^2^, 30 kg/m^2^ ≤ BMI, 5 years ≤ LUTS duration, and 75 ml ≤ prostate volume were finally selected as independent predictors of early SUI into the multivariate logistics regression model. It was visualized as a concise nomogram with satisfactory discrimination and accuracy in both training and validation sets.

**Conclusions:**

A concise nomogram was developed and validated as a useful clinical tool for predicting early SUI post-EEP.

**Supplementary Information:**

The online version contains supplementary material available at 10.1007/s00345-021-03592-x.

## Introduction

Since the introduction of endoscopic enucleation of the prostate (EEP), related technologies and techniques have advanced rapidly [1]. With the advantages of consistent functional improvement, less blood loss, shorter catheterization duration and hospital stay, EEP is gradually becoming a preferred alternative to traditional transurethral resection of the prostate (TURP), especially for patients with sizable prostates [2].

However, stress urinary incontinence (SUI), which occurs in less than 3% of patients after TURP, is reported to represent from 4.1 to 26.0% at 3 months and 0.5 to 3.0% at 12-month follow-up after holmium Laser enucleation of the prostate (HoLEP) [3–7]. Transient urethral dilatation of prostate apex upon enucleation may cause external sphincteric damage. The complete removal of prostate adenoma leads to incomplete closure over the bladder neck and prostatic fossa, mainly contributing to early SUI post-EEP. Additionally, patients' characteristics, physicians' surgical experience and technique were significantly associated with SUI [8, 9]. Although temporary and recoverable, postoperative SUI negatively impacts patients' quality of life outcomes [10].

SUI has been discussed extensively concerning radical prostatectomy (RP). A predictive model for SUI following RP was shown useful for assessing SUI risk preoperatively and guiding timely interventions [11, 12]. However, to date, no comprehensive post-EEP predictive nomogram for SUI has been reported. The early achievement of complete recovery continence (RC) after surgery may also be one of the BPH patients' primary concerns.

In this study, we aimed to develop and validate a concise nomogram to predict the probability of SUI post-EEP. By applying that nomogram, surgeons might screen out and timely intervene in physiology and psychology for the patients at high-risk of SUI.

## Methods

### Study design and patients

The clinical records of 504 patients who underwent plasmakinetic- or diode-based EEP from Mar 2016 to Dec 2019 at Beijing Friendship Hospital (Beijing, China) were reviewed. Our institutional Medical Ethics Committee approved this study, and all patients provided informed consent. Patients with a preoperative history of any urinary incontinence (*n* = 27), urethral stricture (*n* = 4), incidentally discovered prostate cancer (*n* = 6), conversion to TURP (*n* = 3), or missing data (*n* = 6) were excluded.

### Surgical procedure

All plasmakinetic enucleation of the prostate (PKEP) and diode laser enucleation of the prostate (DiLEP) were performed by a single urologist (Dr. Song) in our institution. The PlasmaKinetic SuperPulse System (Gyrus Medical, Cardiff, United Kingdom) was used with a cutting power of 160 W and a coagulating power of 80 W for PKEP. A pulsed 980 nm diode laser was used at 110 W and 10 W for vaporization and coagulation for DiLEP, respectively. First, bluntly dissect the urethral mucosa close to the verumontanum to develop the "true" surgical plane. Keep in mind that, no matter in PKEP or DiLEP, the enucleation procedure was mainly accomplished by the blunt dissection of the transitional prostatic adenoma from the surgical capsule with the endoscopic sheath. The three-lobe technique was then adopted with the urethral mucosa incised to the surgical plane, starting from the bladder neck at 5′o clock, 7′o clock, and 12′ o clock positions anterogradely towards the prostatic apex, but not extend beyond the level of verumontanum. Lastly, the middle lobe was enucleated retrogradely off the bladder neck, followed by the lateral lobes moving clockwise (right lateral lobe) or counterclockwise (left lateral lobe). Morcellation was completed using a rigid offset nephroscope and a Lumenis Versa Cut morcellator system (Lumenis, Yokneam, Israel). Pelvic floor muscle exercise (PFME) was included in the perioperative health education for all patients, who were directed to exercise after removing the urethral catheter approximately 2 days after surgery, and then discharged.

### Variables and outcomes

Baseline and perioperative characteristics included demographics, body mass index (BMI), lower urinary tract symptoms (LUTS) duration, comorbidities including hypertension, diabetes, and hyperlipidemia, prostate volume on the transrectal ultrasound (PV), and total prostate-specific antigen (TPSA). Intra- and postoperative outcomes included catheterization status at the time of surgery, surgery type, operation time (OT), the percent decrease in HGB, and catheterization time. All patients were in a routine visit follow-up at 1 week after the surgery. Those with any urinary leakage with coughing, sneezing, or exertion standing with a full bladder were determined as early SUI. Pad tests were not routinely performed for all patients. Urodynamic assessments were only performed for patients with persistent stress incontinence (> 24 weeks).

### Statistical analysis

Categorical variables were presented as frequency (proportion), and chi-square or Fisher's exact tests were adopted for comparisons. Univariate and multivariate logistic regression analyses were employed to screen out the independent predictive factors for early SUI. The nomogram performance was evaluated by ROC analyses and calibration curves for the training and validation sets. All data management and statistical analyses were carried out using Stata 15.1 for Windows (Stata Corp LLC, College Station, TX). Figures were drawn with R-Studio, Version 1.2.5042 for Windows (Version 1.2.5042, R-Studio, Inc.). All tests were two sided and statistical significance was defined as *P* ≤ 0.05.

## Results

### Patient characteristics

A total of 458 eligible patients seen from Mar 2016 to Dec 2019 were included in the analysis and randomly split at a 7:3 ratio. Detailed characteristics for the whole cohort are shown in Table-Sup. According to the absence or presence of early SUI, two subgroups were divided from the training set and compared on the preoperative and intraoperative parameters. As shown in Table 1, 78 of 326 patients (23.9%) were diagnosed with SUI at 1-week follow-up. Age (*P* < 0.001), BMI (*P* < 0.001), LUTS duration (*P* = 0.004), PV (*P* < 0.001), and OT (*P* = 0.012) all showed statistically significant differences between the two subgroups.

**Table 1:** Baseline demographics and perioperative characteristics of patients by subgroup with or without SUI on training set

Characteristic	SUI		*P* value
	Negative	Positive	
	248 (76.1)	78 (23.9)	
Age, years			** < 0.001**
< 65	81 (32.6)	10 (12.82)	
≤ 65, < 70	111 (44.76)	34 (43.59)	
≥ 70	56 (22.58)	34 (43.59)	
BMI, kg/m^2^			** < 0.001**
< 25	137 (55.3)	29 (37.2)	
≤ 25, < 30	103 (41.5)	38 (48.7)	
≥ 30	8 (3.2)	11 (14.1)	
LUTS duration, years			**0.004**
< 5	156 (62.9)	34 (43.6)	
≥ 5	92 (37.1)	44 (56.4)	
Hypertension, *n* (%)	107 (43.1)	42 (53.8)	0.118
Hyperlipidemia, *n* (%)	47 (19.0)	19 (24.4)	0.333
Diabetes, *n* (%)	55 (22.2)	20 (25.6)	0.539
Prostate volume, ml			** < 0.001**
< 75	185 (74.6)	35 (44.9)	
≥ 75	63 (25.4)	43 (55.1)	
Total PSA, ng/ml			0.242
< 4	132 (53.2)	35 (44.9)	
≥ 4	116 (46.8)	43 (55.1)	
Catheterization at surgery, *n* (%)	76 (30.6)	33 (42.3)	0.073
Type of surgery, *n* (%)			0.437
PKEP	131(52.8)	37(47.4)	
DiLEP	117(47.2)	41(52.6)	
Operation time, min			**0.012**
< 100	142 (57.3)	32 (41.0)	
≥ 100	106 (42.7)	46 (59.9)	
Decrease in HGB, %			0.784
< 5	86 (34.7)	25 (32.1)	
≥ 5	162 (65.3)	53 (67.9)	
Catheterization duration, days			0.297
< 3	106 (42.7)	39 (50.0)	
≥ 3	142 (57.3)	39 (50.0)	

The significant statistical differences were emphasized in a bold version

### Determination of independent predictors for postoperative SUI

The following significant variables from the univariate analysis were further analyzed in the multivariate model, including forward, backward, and stepwise regression analyses: 65 years ≤ age < 70 years (2.48, 1.20–5.56, *P* = 0.019), 75 years ≤ age (4.92, 2.32–11.25, *P* < 0.001), 25 kg/m^2^ ≤ BMI < 30 kg/m^2^ (1.74, 1.01–3.03, *P* = 0.005), 30 kg/m^2^ ≤ BMI (6.50, 2.42–18.18, *P* < 0.001), 5 years ≤ LUTS duration (2.19, 1.31–3.70, *P* < 0.001), 75 ml ≤ prostate volume (3.61, 2.13–6.16, *P* < 0.001) and 100 min ≤ surgery time (1.93, 1.15–3.25, *P* = 0.013). Finally, as shown in Table 2, the following independent predictors for early SUI were incorporated into the multivariate logistics regression model: 65 years ≤ age < 70 years (OR, 95%CI: 2.43, 1.11–5.75, *P* = 0.032), 75 years ≤ age (5.20, 2.29–12.75, *P* < 0.001), 25 kg/m2 ≤ BMI < 30 kg/m^2^ (1.99, 1.10–3.68, *P* = 0.025), 30 kg/m2 ≤ BMI (6.60, 2.22–20.58, *P* < 0.001), 5 years ≤ LUTS duration (2.37, 1.34–4.24, *P* = 0.003) and 75 ml ≤ prostate volume (6.37, 2.57–17.80, *P* < 0.001).

**Table 2:** Univariate and multivariable logistic regression analysis on training set

Variables	Univariate			Multivariate		
	OR	95% CI	*P* value	OR	95% CI	*P* value
Age, years						
< 65	1.00	–	–	1.00	–	–
≤ 65, < 70	2.48	1.20–5.56	**0.019**	2.43	1.11–5.75	**0.032**
≥ 70	4.92	2.32–11.25	** < 0.001**	5.20	2.29–12.75	** < 0.001**
BMI, kg/m^2^						
< 25	1.00	–	–	1.00	–	–
≤ 25, < 30	1.74	1.01–3.03	**0.005**	1.99	1.10–3.68	**0.025**
≥ 30	6.50	2.42–18.18	** < 0.001**	6.60	2.22–20.58	** < 0.001**
LUTS duration, years						
< 5	1.00	–	–	1.00	–	–
≥ 5	2.19	1.31–3.70	** < 0.001**	2.37	1.34–4.24	**0.003**
Prostate volume, ml						
< 75	1.00	–	–	1.00	–	–
≥ 75	3.61	2.13–6.16	** < 0.001**	6.37	2.57–17.80	** < 0.001**
Operation time, mins						
< 100	1.00	–	–	1.00	–	–
≥ 100	1.93	1.15–3.25	0.013	0.44	0.16–1.11	0.091

The significant statistical differences were emphasized in a bold version

### Construction and validation of the nomogram

**Fig. 1:**
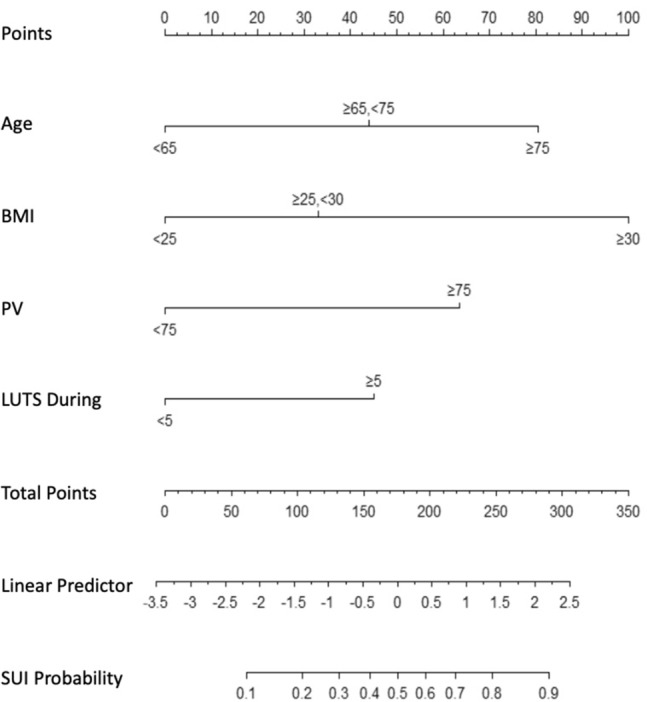
Nomogram for predicting the onset of early stress urinary incontinence (SUI) following endoscopic enucleation of the prostate (EEP)

**Fig. 2:**
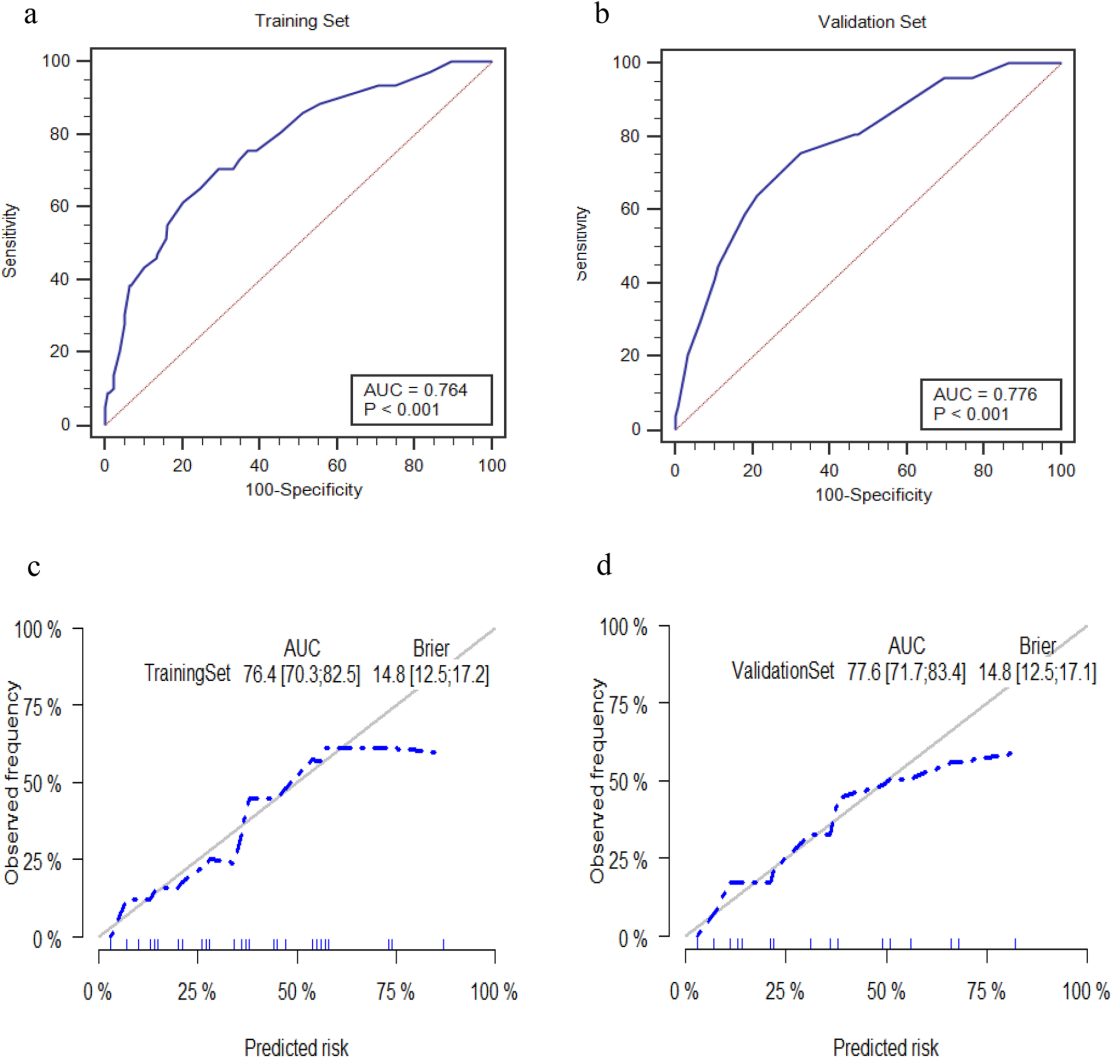
Receiver operating characteristic (ROC) and calibration curves for predicting early stress urinary incontinence (SUI) following endoscopic enucleation of the prostate (EEP) on the training set (**a** and **c**) and validation set (**b** and **d**)

The multivariate logistic regression model was visualized as a concise nomogram (Fig. 1). Performance in predicting early SUI onset was evaluated using the area under the curve (AUC) of the receiver operating characteristic (ROC) analysis and calibration curves. The AUC was 0.764 (95%CI 0.703–0.825) and 0.775 (95%CI 0.716–0.833) in the training and validation sets, respectively (Fig. 2a and b), indicating satisfactory discrimination. Consistency between the calibration curve and the 45-degree ideal line reflected adequate prediction accuracy by nomogram and actual SUI in both the training and validation sets (Fig. 2c and d).

## Discussion

Endoscopic enucleation of the prostate (EEP) can achieve comparable urinary function improvement with lower complication rates than TURP [2, 13]. However, a relatively high proportion of patients develop postoperative SUI, impacting their satisfaction and QoL. Thus, SUI deserves further investigations and early interventions to improve patients' quality of life after surgery.

Some clinical baseline and perioperative data are reported to predict the probability of developing SUI after prostate surgery. Age is an established risk factor for SUI at 1, 3, and 12 months after robot-assisted RP [12]. Similarly, increasing age is positively correlated with the occurrence of transient UI or SUI in transurethral vaporization of the prostate (TUVP), transurethral enucleation with bipolar (TUEB), or HoLEP [4, 7, 14]. Elevated BMI and enlarged prostate volume are also linked with postoperative SUI [15]. Transient stretching of the external sphincter and incomplete closure over the bladder neck and prostatic fossa resulting from complete surgical removal of enlarged prostate adenomas, combined with obesity-associated increased abdominal pressure, increases the probability of SUI [8, 9, 16]. In a retrospective analysis of 949 consecutive patients treated with HoLEP by a single surgeon within 10 years, Elmansy et al. confirm prostate volume greater than 81gm as a statistically significant factor for postoperative SUI (*P* < 0.02) [17]. Recently, Xu et al. reports that age ≥ 70 years (OR:9.239; 95%CI 4.616–18.495; *P* < 0.001) and prostate volume ≥ 90 ml on transrectal ultrasound (OR:15.390; 95% CI 8.077–29.326; *P* < 0.001) were significant factors for developing SUI after PKEP [18]. Consistent with these results, the present study found age, BMI, and prostate volume to be independent predictors for the onset of SUI after EEP in multivariate analyses. Besides, we found that LUTS duration ≥ 5 years negatively affected patient RC. LUTS is a progressive, age-related, nonorganic-specific group of symptoms [19, 20], and to date, there have been no reports on the association between LUTS duration and SUI occurrence. Age, BMI, metabolic syndrome, and pelvic floor muscle functional abnormalities contribute more to the initiation and persistence of LUTS than prostate volume or bladder dysfunction [21, 22]. Considering these multifactorial etiologies, LUTS may represent a comprehensive predictive factor for SUI or mixed urinary incontinence (MUI) after surgery.

Longer operation time means a longer duration of damage to the inner longitudinal layer around the apical gland and external sphincter [8, 17]. Nam et al. report that total operation time (OR = 3.849; 95% CI = 1.613∼9.185; *P* = 0.002) is associated with UI occurrence within 3 months after HoLEP [4]. Kobayashi et al. reports enucleation time > 100 min (OR, 2.54; 95% CI 1.03–6.30; *P* = 0.043) to be an independent and significant predictor of postoperative UI, but not SUI [23]. Considering that operating time increases with increasing prostate volume, we also performed a Spearman correlation analysis between operation time ≥ 100 min and prostate volume ≥ 75 ml (*P* < 0.001). This relevance accounts for the fact that a total operation time ≥ 100 min was not shown to be a significant factor based on multivariate analysis. Diabetes, catheterization status at surgery, and decreasing PSA percentage were also shown as independent factors for SUI [17, 24], but their significance was not confirmed in our analyses.

Regarding SUI following RP, the predictive nomograms can help physicians to assess individual risk of developing SUI and conduct timely, perioperative incontinence interventions. Hirasawa et al. created a nomogram that only consists of age and prostate volume, developed from a retrospective analysis of data for 584 patients who underwent TUEB to predict postoperative transient UI [7]. However, in this study, the type of UI, such as stress, urgency, or mixed UI, was not assessed. Some important patient characteristics that are potential predictive factors, such as BMI and medical history, were none involved. Although a C-index of 0.690 cannot be considered a bad standard for models, its availability lacked validation in an additional test set. This is a notable limitation that restricts the application to populations outside of the study cohort [25].

Thus, to provide a convenient, individualized, and effective method for predicting early SUI following EEP, we developed and validated a nomogram using baseline and perioperative data from 458 patients in our institution. Considering that a concise nomogram with simple and intuitive features is easier to interpret, continuous variables were categorized and then incorporated into the model via crude univariate analysis and multivariate analysis selection procedures. The latter included forward, backward, and stepwise regression analysis [26]. Ultimately, four features were incorporated into our nomogram.

The area under the curve was used to assess nomogram performance and found to be 0.764 (95%CI 0.703–0.825) and 0.775 (95%CI 0.716–0.833) in the training and validation sets, respectively. An adequate consistency between the calibration curve and the 45-degree ideal line was shown in the calibration analysis on both sets.

Taken together, we developed and validated a tool for predicting early postoperative SUI in patients with BPH who have undergone either PKEP or DiLEP. This is the first report on a nomogram to predict SUI probability following EEP to the best of our knowledge. Based on the satisfactory accuracy and discrimination outcomes, physicians can easily adopt the nomogram to screen out the patients with high-risk of SUI. Individualized consultations before surgery could reduce anxiety. Moreover, starting PFME before and continuing it after surgery could facilitate RC [27–29].

Several limitations to the study findings should be considered. First, our conclusion was based on retrospective data analyses with the total number of patients was limited. Second, there are no widely accepted standard criteria defining postoperative SUI. As a result, the reported SUI incidence after EEP varies widely, from 4.1–26.0% at 3 months and 0.5–3.0% at 12 months follow-up [3–7]. In our study, SUI was defined as any urinary leakage when coughing, sneezing, or exertion by standing with a full bladder. However, the data lacked objective evaluation by routine pad test or postoperative urodynamic study. The incidence of SUI in the training set was 4.3% and 0.2% at 3- and 12-month follow-up, respectively, similar to previously reported findings. Third, the study involved two types of surgery, PKEP and DiLEP, and this heterogeneity may have affected the perioperative data. However, our previous study (not published) found no statistical difference between PKEP and DiLEP in terms of impact on postoperative SUI, which is consistent with the conclusion of a recent meta-analysis [30]. Fourth, all interventions were performed by a single surgeon, so the effect of surgical experience on postoperative SUI, as previously reported, could not be evaluated [3, 23]. Accordingly, a model integrated learning-curve variable will be considered in the design of future studies. Finally, our tool was developed and validated at the same institution and would be enhanced by external validation with cohort data from different institutions. Fortunately, we have started collecting data from multiple health centers in China for this purpose.

## Conclusions

We developed and validated a concise nomogram with satisfying accuracy and discrimination for individual prediction of early SUI after transurethral anatomical enucleation of the prostate. Although the specific surgical experience and technique involved in the nomogram need to be stressed, its application could help urologists to service patients with better counseling and timely interventions preoperatively.

## Supplementary Information

Below is the link to the electronic supplementary material.

Supplementary file1 (DOCX 20 KB)
