# Supplementary material for: Development and validation of a nomogram for predicting early stress urinary incontinence following endoscopic enucleation of the prostate
Source: World J Urol. 2021 Jan 21;39(9):3447–53. doi: 10.1007/s00345-021-03592-x (PMC8510930; doi:10.1007/s00345-021-03592-x)
Supplement: Supplementary file 1 — Supplementary file1 (DOCX 20 KB) [file 345_2021_3592_MOESM1_ESM.docx]

**Table-Sup.:** Baseline demographics and perioperative characteristics of patients in the training and validation set.

| Characteristic | Total set | Training set Validation set | | P-Value |
| --- | --- | --- | --- | --- |
| n (%) | 458 (100) | 326 (71.3) | 132 (28.7) |  |
| Age, years |  |  |  | 0.765 |
| <65 | 124(27.1) | 91(27.9) | 33(25.0) |  |
| ≤65, <70 | 204(44.5) | 145(44.5) | 59(44.7) |  |
| ≥70 | 130(28.4) | 90(27.6) | 40(30.3) |  |
| BMI, kg/m^2^ |  |  |  | 0.784 |
| <25 | 235(51.3) | 166(50.9) | 71(53.8) |  |
| ≤25, <30 | 198(43.2) | 141(43.3) | 55(41.7) |  |
| ≥30 | 25(5.5) | 19(5.8) | 6(4.5) |  |
| LUTS duration, years |  |  |  | 0.468 |
| <5 | 262(57.2) | 190(58.3) | 72(54.5) |  |
| ≥5 | 196(42.8) | 136(41.7) | 60(45.5) |  |
| Hypertension , n(%) | 212 (46.3) | 149(45.7) | 64 (48.5) | 0.606 |
| Hyperlipidemia , n(%) | 96 (21.0) | 66(20.3) | 30 (22.7) | 0.612 |
| Diabetes , n(%) | 109 (23.8) | 75(23.0) | 34 (25.8) | 0.546 |
| Prostate volume, mL |  |  |  | 0.505 |
| <75 | 228(49.8) | 220(67.5) | 94(71.2) |  |
| ≥75 | 230(50.2) | 106(32.5) | 38(28.8) |  |
| Total PSA, ng/mL |  |  |  | 0.302 |
| <4 | 216(47.2) | 167(51.2) | 60 (45.5) |  |
| ≥4 | 242(52.8) | 159(48.8) | 72 (54.5) |  |
| Catheterization  at surgery, n(%) | 158(34.5) | 109(33.4) | 49(37.1) | 0.450 |
| Type of surgery |  |  |  | 0.606 |
| PKEP | 240(52.4) | 168(51.5) | 72(54.5) |  |
| DiLEP | 218(49.6) | 158(48.5) | 60(45.5) |  |
| Operation time, mins |  |  |  | 0.535 |
| <100 | 233(50.9) | 174 (53.4) | 76(56.8) |  |
| ≥100 | 225(49.1) | 152 (46.6) | 56(43.2) |  |
| Decrease in HGB, % |  |  |  | 0.389 |
| <5 | 162 (35.4) | 111(34.1) | 51 (38.6) |  |
| ≥5 | 296 (64.6) | 215(65.9) | 81 (61.4) |  |
| Catheterization duration, days |  |  |  | 0.756 |
| <3 | 206 (45.0) | 145(44.5) | 61 (46.2) |  |
| ≥3 | 252 (55.0) | 181(55.5) | 71 (53.8) |  |
